# How Is Vaccine Effectiveness Scaled by the Transmission Dynamics of Interacting Pathogen Strains with Cross-Protective Immunity?

**DOI:** 10.1371/journal.pone.0050751

**Published:** 2012-11-30

**Authors:** Ryosuke Omori, Benjamin J. Cowling, Hiroshi Nishiura

**Affiliations:** 1 School of Public Health, The University of Hong Kong, Hong Kong, Hong Kong SAR, People’s Republic of China; 2 PRESTO (Precursory Research for Embryonic Science and Technology), Japan Science and Technology Agency, Kawaguchi, Saitama, Japan; National Institutes of Health, United States of America

## Abstract

**Background:**

Many novel vaccines can cover only a fraction of all antigenic types of a pathogen. Vaccine effectiveness (VE) in the presence of interactions between vaccine strains and others is complicated by the interacting transmission dynamics among all strains. The present study investigated how the VE estimates measured in the field, based on estimated odds ratio or relative risks, are scaled by vaccination coverage and the transmission dynamics in the presence of cross-protective immunity between two strains, i.e. vaccine and non-vaccine strains.

**Methodology/Principal Findings:**

Two different types of epidemiological models, i.e. with and without re-infection by the same antigenic type, were investigated. We computed the relative risk of infection and the odds ratio of vaccination, the latter of which has been measured by indirect cohort method as applied to vaccine effectiveness study of *Streptococcus pneumoniae*. The VE based on the relative risk was less sensitive to epidemiological dynamics such as cross-protective immunity and vaccination coverage than the VE calculated from the odds ratio, and this was especially the case for the model without re-infection. Vaccine-induced (cross-protective) immunity against a non-vaccine strain appeared to yield the highest impact on the VE estimate calculated from the odds ratio of vaccination.

**Conclusion:**

It is essential to understand the transmission dynamics of non-vaccine strains so that epidemiological methods can appropriately measure both the direct and indirect population impact of vaccination. For pathogens with interacting antigenic types, the most valid estimates of VE, that are unlikely to be biased by the transmission dynamics, may be obtained from longitudinal prospective studies that permit estimation of the VE based on the relative risk of infection among vaccinated compared to unvaccinated individuals.

## Introduction

Since new vaccines are continuously developed and introduced to human population, it is essential to assess how effective the vaccination is both at the individual and population levels. Conventionally, vaccine efficacy at an individual level has been measured as the relative reduction in the conditional risk of infection (given an exposure) among vaccinated individuals as compared to unvaccinated, most typically, through randomized controlled trials. To make a clear distinction in the present study, hereafter the vaccine effectiveness (VE) is defined as the relative reduction of the risk of infection among vaccinated population to that among unvaccinated population at a population level, reflecting herd immunity and other properties of the transmission dynamics [1]. Since the latter measure, i.e., the risk at the population level, involves an observational problem that stems from dependence in the risk of infection between individuals in the same population, epidemiological studies of vaccination have required us to consider various types of study designs and statistical methods [2]. Moreover, due to the dependence in the risk of infection, vaccine effectiveness at the population level is likely to differ from vaccine efficacy at an individual level, and thus, it has been shown that the vaccine effectiveness is influenced not only by the efficacy but also by vaccination coverage, contact patterns, diagnostic performance and other factors that govern the transmission dynamics of infectious diseases [3]. The pressing public health question of identifying such epidemiological determinants of vaccine effectiveness and corresponding selection of appropriate study designs have been addressed by employing epidemiological modeling techniques [1], [4].

However, two critical aspects of vaccination have yet to be explored. First, published epidemiological studies that linked efficacy with effectiveness by employing mathematical models have tended to focus on an epidemic setting [1], while in reality a newly introduced vaccine tends to target an existing endemic disease. Rather than an epidemic model that does not account for underlying demographic dynamics in the human population, one has to consider epidemiological dynamics on a longer time scale. Second, many recent vaccines are designed to prevent infection with a pathogen that consists of multiple antigenic types, and thus, it can be important to explicitly model the epidemiological dynamics of two or more interacting strains to capture the underlying epidemiological mechanisms.

When it comes to multiple antigenic types, it should be noted that many diseases are caused by antigenically diverse pathogens, and more importantly, many new vaccines can only protect against infection with particular antigenic types. In such an instance, epidemiology of vaccination against a multi-strain disease involves not only the issue of limited clinical protection from a limited number of strains but also a complex epidemiological problem in scaling vaccine effectiveness by vaccination coverage and the transmission dynamics. In the absence of vaccination, susceptibility to a certain strain is determined not only by the exposure to the same strain, but also by exposure to other strains that offer cross-protective immunity against the strain of interest. In that case, vaccination can not only directly reduce the risk of infection with vaccine strain(s) but also indirectly vary the risk of infection by varying cross-protective immunity that is induced by infection with other strains. Thus, the effectiveness of vaccination in the presence of cross-protective immunity between vaccine strains and others is characterized by complex interactions among all strains [5], [6], [7], [8].

The present study aims to clarify how the epidemiologically observed risk measures, which are used to inform vaccine effectiveness, in the presence of interacting antigenic types, are scaled by vaccination coverage and the transmission dynamics. Employing simple epidemiological models that are suited to consider an endemic situation, we identify key epidemiological determinants that influence the vaccine effectiveness and discuss the current data gaps that need to be addressed to appropriately assess the effectiveness of vaccination.

## Materials and Methods

### Diseases with and without Re-infection

**Figure 1:**
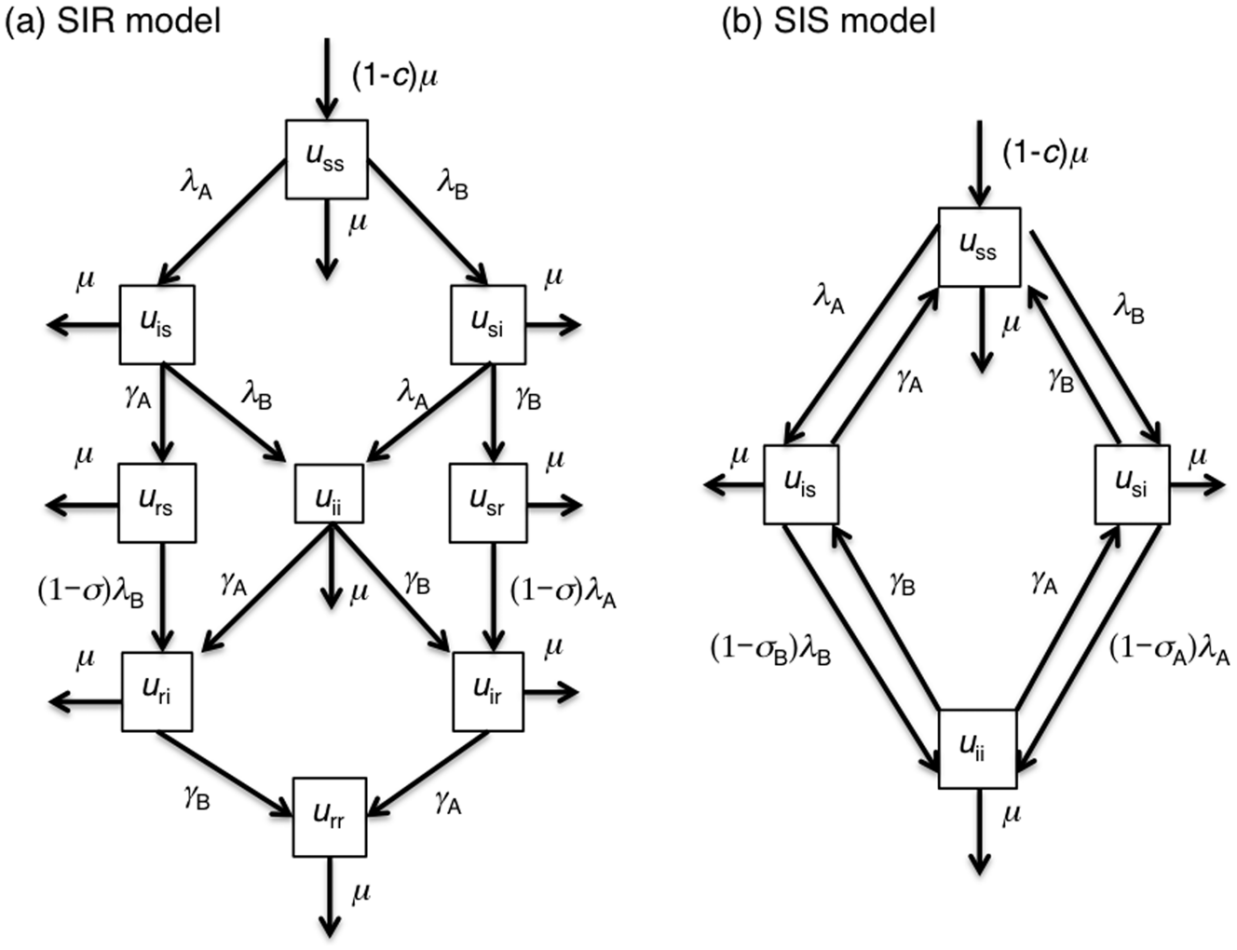
Transmission dynamics of two-strain disease in the presence of cross-protective immunity. (a) Model without re-infection with an identical antigenic type (SIR-type; susceptible-infectious-recovered) and (b) model with re-infections by an identical antigenic type (SIS-type; susceptible-infected-susceptible). SIR model is intended to capture the epidemiological dynamics of EV71, while SIS model is applied to pneumococcus. [Compartments] Variable *u* represents unvaccinated. Two subscripts represent the state of infection (or carriage) with respect to VT and NVT, respectively. For example, *u*_si_ represents unvaccinated host who is susceptible to VT but is infected with NVT. [Parameters] *c*, vaccination coverage; *μ*, background birth and death rates of human host; *λ*_A_ and *λ*_B_, the rates of infection with VT (vaccine type) and NVT (non-vaccine type), respectively; *γ*_A_ and *γ*_B_, recovery rates from infection with VT and NVT, respectively; *σ*, the relative reduction of the risk of infection upon exposure by cross-protective immunity in the SIR model; *σ*_A_ and *σ*_B_, the relative reduction of the risk of carriage acquisition upon exposure to VT and NVT by competition, respectively, in the SIS model. For simplicity, both panels represent the population dynamics of unvaccinated population alone. In case no vaccination takes place, *c* is equal to 0.

Hereafter, we refer to antigenic types that are covered by vaccination as vaccine type (VT) and others as non-vaccine types (NVT). For simplicity, the present study considers a situation in which there are only two strains, i.e., VT and NVT. For the exposition of our model-based arguments, we consider two diseases with multiple antigenic types. One is hand-foot-and-mouth disease (HFMD), especially that caused by enterovirus 71 (EV71). It has been demonstrated that EV71 involves genogroups with cross-antigenicity in the experimental setting [9], while a vaccine under development may only partially protect vaccinated individuals from certain antigenic type(s) [10]. In this setting, we assume that infection with a single strain (either VT or NVT) elicits life-long immunity, and thus, we model the epidemiological dynamics by employing the SIR (Susceptible-Infectious-Recovered) model with two strains, but without re-infection (Figure 1).

Another disease to be considered is the infection, carriage and colonization with *Streptococcus pneumoniae* (pneumococcus) which involves more than 90 serotypes, while currently available vaccines have covered only a certain number of serotypes (e.g. PCV7 that offers protection from 7 serotypes) [11]. As is also the case for EV71, a relative reduction in the risk of carriage acquisition has been demonstrated for pneumococcus by competition between VT and NVT, and the replacement of major serotypes has been observed after introducing PCV7 into a new community [12]. It is known that the natural remittance occurs, and carriages due to recurrent exposures with the same serotype have also been observed [13]. Thus, we use the SIS (Susceptible-Infected-Susceptible) model that allows multiple re-infections (Figure 1).

### Vaccine Effectiveness

Vaccine effectiveness (VE) is calculated as the relative risk reduction among the vaccinated population as compared with the unvaccinated population [3]. The relative risk (RR) of infection or its approximation can be measured from epidemiological studies. In retrospective studies, the odds ratio (OR) of infection has been employed as an approximation to RR. The OR is often used due to the epidemiological and clinical characteristics of a disease (e.g. a clinically non-apparent disease with low prevalence).

As another type of OR for the estimation of vaccine effectiveness, an indirect cohort method, or the so-called Broome method, has been applied to pneumococcus [14], [15], [16], in which the OR of “vaccination” among VT cases to NVT cases with IPD (invasive pneumococcal disease) has been used for the assessment of vaccine effectiveness. This method has been proposed because the exposure with pneumococcus tends to result in clinically very mild infection or carriage, and perhaps also because the method requires us to collect only the counts of IPD cases with strain information (i.e. VT or NVT) and their vaccination histories [14]. Although the Broome method has been positively assessed elsewhere [11], the validity has yet to be explicitly assessed by accounting for detailed interactions between VT and NVT in a mathematically rigorous manner, because the method inherently assumes that there is no interference between VT and NVT. To examine how vaccine effectiveness is scaled by the coverage, cross-protective immunity and other epidemiological features, we computed the vaccine effectiveness using two different epidemiological measures, using mathematical models with an assumed vaccine efficacy. Vaccine effectiveness calculated from the relative risk of infection, VE_R_ and from the odds ratio of vaccination, VE_O_ are written as
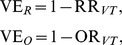
(1)where RR_VT_ is the relative risk of infection with VT among vaccinated individuals as compared with unvaccinated, and OR_VT_ is the odds ratio of vaccination among IPD cases with VT to IPD cases with NVT.

### Mathematical Model

Figure 1 shows the structures of the SIR and SIS type models. In the case of SIR (i.e. model for EV71), we assume that serotype-specific life-long immunity is acquired from infection with either VT or NVT which also yields a cross-protective immunity against infection with the other serotype. Cross-protective immunity is similarly considered in the SIS model (i.e. model for pneumococcus), and this model permits re-infections with the same serotype. We consider two different types of immunity for both naturally acquired and vaccine-induced ones, (i) all-or-nothing type which elicits a perfect protection from infection with probability *σ*, and (ii) leaky type immunity which reduces the conditional probability of infection given an exposure by the factor of relative reduction *σ*. When we adopt either of the two types for vaccine-induced immunity, the naturally acquired immunity is also assumed to follow the same type of immunity. For both models, the background birth and death rates of human host are assumed as identical, *μ*. The mathematical descriptions of these models are given in the Appendix. As a general representation of the two models, we also explore the so-called SIRS (susceptible-infectious-recovered-susceptible) model in the Online Supporting Material.

To assess VE, we use endemic steady state solution of the models in the presence of vaccination. As for the epidemiological actions of different types of vaccine, all-or-nothing vaccine is assumed to provide vaccinated individuals with perfect protection against VT (or NVT) with a probability *ε*_VT_ (or *ε*_NVT_), and the remaining proportion of vaccinated individuals remain susceptible. Leaky vaccine reduces the instantaneous risk of infection upon each exposure to VT (or NVT) by the factor of reduction *ε*_VT_ (or *ε*_NVT_). We specifically consider the two types of vaccine, because the actual biological action of both vaccines is unknown.

**Table 1:** Parameter values for the SIR (Susceptible-Infectious-Recovered) model as applied to the epidemiological dynamics of enterovirus 71.

Symbol	Description	Baseline value	Plausible range inpublished studies	References
*σ*	Cross-protective immunity	0.5		Assumed
*ε*_VT_	Vaccine efficacy against VT	0.6–0.8		Assumed
*ε*_NVT_	Vaccine efficacy against NVT	0.3		Assumed
*R*_0,VT_	The basic reproduction number of VT	4.0	1.4–6.5	[18], [19]
*R*_0,NVT_	The basic reproduction number of NVT	4.0	1.4–6.5	[18], [19]
1/*γ*_A_	Infectious period of VT (days)	7	3–14	[18], [19]
1/*γ*_B_	Infectious period of NVT (days)	7	3–14	[18], [19]

**Table 2:** Parameter values for the SIS (Susceptible-Infected-Susceptible) as applied to the epidemiological dynamics of *Streptococcus pneumoniae.*

Symbol	Description	Baseline value	Plausible range inpublished studies	References
*σ*_A_	Relative reduction in carriage acquisitionof VT by competition	0.1	0.0–0.5	[13]
*σ*_B_	Relative reduction in carriage acquisitionof NVT by competition	0.4	0.0–1.0	[13]
*ε*_VT_	Vaccine efficacy against carriage acquisition of VT	0.6	0.1–0.9	[28] and assumed
*ε*_NVT_	Vaccine efficacy against carriage acquisition of NVT	0.1		Assumed
*ε*_VTi_	Vaccine efficacy against invasion of VT	0.7	0.7–1.0	[13], [29]
*ε*_NVTi_	Vaccine efficacy against invasion of NVT	0.1		Assumed
*R*_0,VT_	The basic reproduction number of VT	1.3	0.9–1.4	[13]
*R*_0,NVT_	The basic reproduction number of NVT	1.2	1.0–1.6	[13]
1/*γ*_A_	Infectious period of VT (days)	70	40–120	[13]
1/*γ*_B_	Infectious period of NVT (days)	50	40–120	[13]

**Figure 2:**
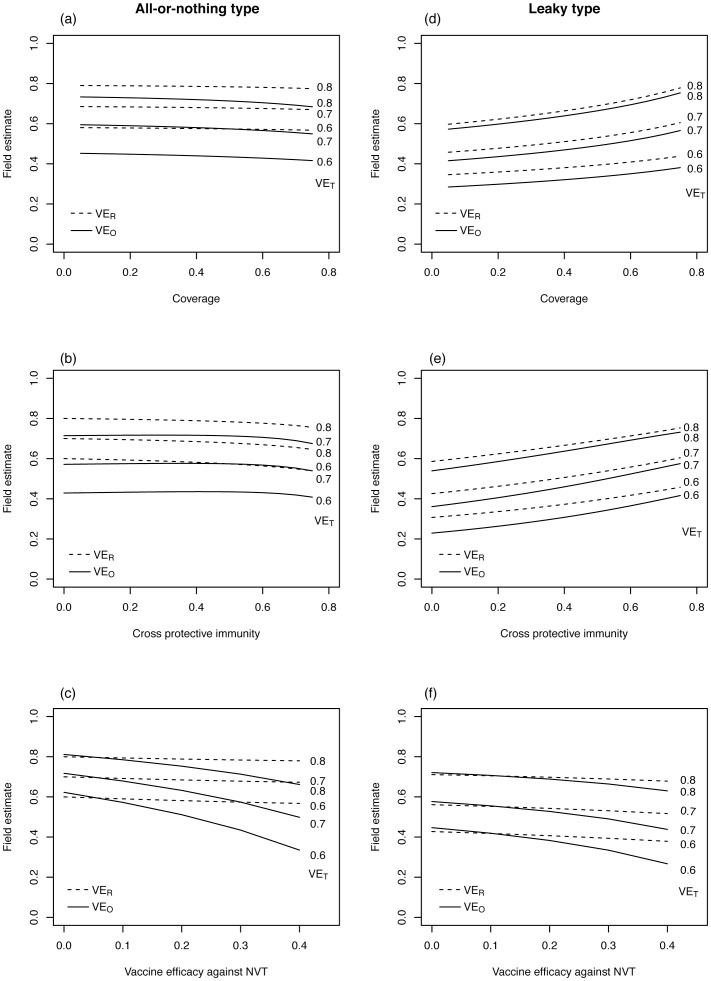
Vaccine effectiveness in the SIR (Susceptible-Infectious-Recovered) model. Field estimate (vertical axis) represents the vaccine effectiveness estimate derived from empirical observation in the field. Solid line represents vaccine effectiveness based on odds ratio, VE_O_, while broken line represents that based on relative risk, VE_R_. Assumed vaccine efficacy against VT (vaccine type) is shown at the right end of each line. Cross-protective immunity is expressed as perfect protection with a probability *σ* for an all-or-nothing type vaccine, and expressed as the relative reduction in the instantaneous risk of infection upon exposure against a serotype among those who have already experienced infection with the other serotype for a leaky type vaccine. (a)-(c) show the effectiveness of all-or-nothing vaccine (i.e. perfect protection given successful immunization and no protection for unsuccessful vaccination), whereas (d)-(f) show the effectiveness of leaky vaccine (i.e. imperfect protection for all vaccinated individuals).

**Figure 3:**
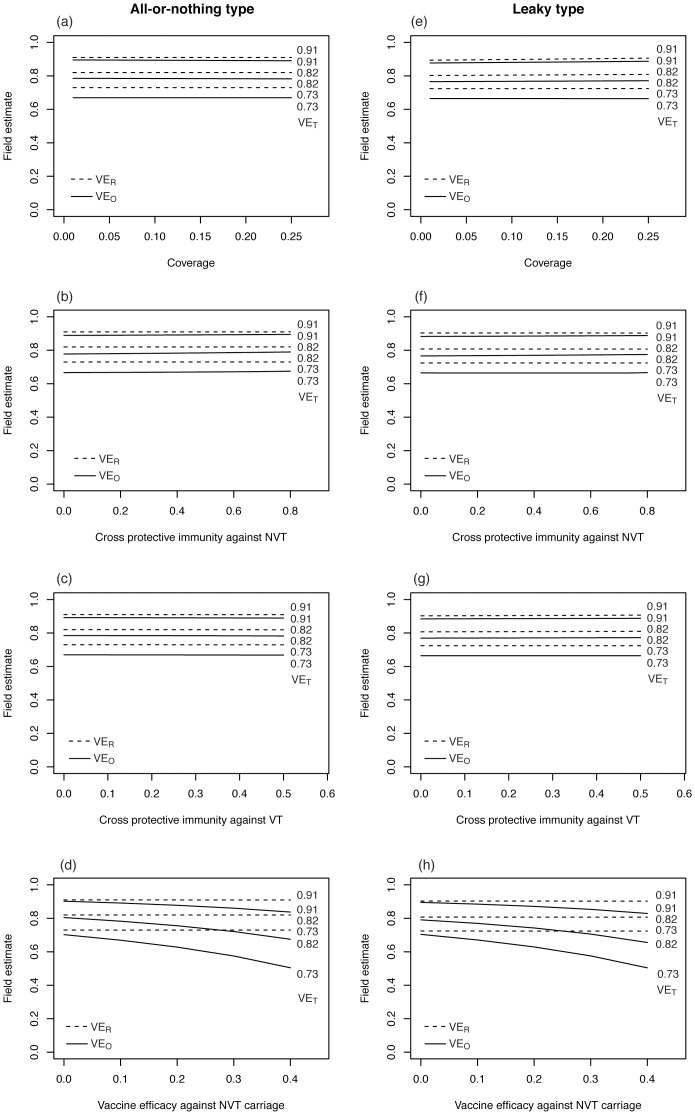
Vaccine effectiveness in the SIS (Susceptible-Infected-Susceptible) model. Field estimate (vertical axis) represents the vaccine effectiveness estimate derived from empirical observation in the field. Solid line represents vaccine effectiveness based on odds ratio, VE_O_, while broken line represents that based on relative risk, VE_R_. Assumed vaccine efficacy against VT (vaccine type) is shown at the right end of each line. Vaccine-induced immunity was dealt with as in two different ways, (i) all-or-nothing type or (ii) leaky type. (a)-(d) show the effectiveness of all-or-nothing vaccine (i.e. perfect protection given successful immunization and no protection for unsuccessful vaccination), whereas (e)-(h) show the effectiveness of leaky vaccine (i.e. imperfect protection for all vaccinated individuals).

#### All-or-nothing vaccine

Analyzing the SIR model in an endemic steady state, vaccine effectiveness against VT, given vaccine efficacy against VT, *ε*_VT_, using the relative risk of infection is calculated as
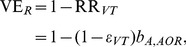
(2)where, as described in the Supporting Information, *b*_A,AOR_ adjusts the interaction between VT and NVT and herd immunity, i.e.,
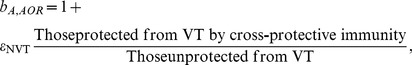
(3)where εNVT is the vaccine efficacy against NVT. Here “those protected from VT by cross-protective immunity” represents the population fraction of individuals who escape from infection with VT due to cross-protective immunity (or the frequency of those escaping from carriage acquisition of VT due to competition between VT and NVT). The mathematical representation of these terms and the derivation are given in the Supporting Information. Using odds ratio of vaccination, VE_O_ is
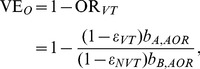
(4)where, again as described in the Supporting Information, *b*_B,AOR_ adjusts the interaction between NVT and VT and the transmission dynamics, as in a similar fashion to *b*_A,AOR_ and reads



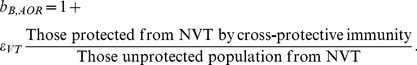
(5)In the absence of cross-protective immunity as well as vaccine-induced protection against NVT, VE_R_ is equal to an unbiased vaccine efficacy against VT, *ε*_VT_. Since equation (3) is always 1 or greater, VE_R_ tends to be an underestimate of *ε*_VT_. Moreover, if additionally there is no cross-protective immunity between VT and NVT and if vaccine efficacy against NVT is zero, VE_O_ is equal to both VE_R_ and *ε*_VT_.

#### Leaky vaccine

At an endemic steady state of SIR model, vaccine effectiveness against VT is calculated using the relative risk of infection:
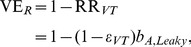
(6)where *ε*_VT_ is the unbiased vaccine efficacy against VT. *b*_A,Leaky_ scales the impact of interaction and herd immunity on the epidemiological dynamics:
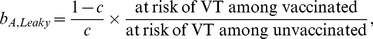
(7)where c denotes the effective vaccination coverage (i.e. the fraction of vaccinated and protected fraction). VE_O_ is written as
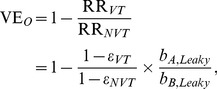
(8)where *b*_B,Leaky_ is



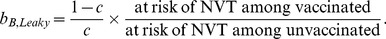
(9)From equations (6) and (7), VE_R_ is equal to *ε*_VT_ if the frequency of those at risk of VT among vaccinated is equal to the frequency of those at risk of VT among unvaccinated. Unlike all-or-nothing vaccine, VE_R_ is unequal to *ε*_VT_ even when there is no cross-protective immunity, because the size of the at risk population depends on vaccination coverage and vaccine efficacy *ε*_VT_. In the absence of cross-protective immunity and given that vaccine efficacy against NVT is zero, VE_O_ is equal to VE_R_. The arguments similar to equations (2)-(9) can also hold for the SIS model (see Supporting Information).

Only for pneumococcus, it should be noted that we assess the vaccine effectiveness by the reduced incidence of IPD cases. We separate the efficacy of pneumococcal vaccine into two parts, i.e. the efficacy against becoming a carriage of VT, *ε*_VT_, and conditional protection from IPD given infectious exposure, *ε*_VTi_. The unconditional vaccine efficacy against IPD caused by VT and NVT, *ε*_1_ and *ε*_2_, is written as
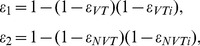
(10)where *ε*_NVT_ and *ε*_NVTi_ are the efficacy against NVT and conditional protection from IPD given an infectious exposure, respectively.

#### Heterogeneity

Although the above mentioned descriptions rest on the assumption of homogeneous mixing between vaccinated and unvaccinated individuals, it may be more realistic to consider heterogeneous contact patterns, e.g. more frequent contact within the unvaccinated subpopulation. To describe within- and between-group transmission in a population that consists of vaccinated and unvaccinated individuals, we employ the so-called preferred mixing assumption [17]. The transmission coefficient of serotype *h* (VT or NVT) that a primary case in subpopulation *i* transmits to susceptibles in subpopulation *j*, *β*_hij_ is proportional to
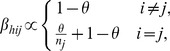
(11)where *n*_j_ represents the relative population size of subpopulation *j* and *θ* is the assortativity coefficient, i.e., the proportion of contacts that are spent for within-subpopulation mixing. As the sensitivity analysis, we vary *θ* from 0–0.3 and examine the sensitivity of vaccine effectiveness to *θ*.

Tables 1 and 2 show parameter values and ranges that we examine in numerical analysis. The sensitivity of VE_R_ and VE_O_ to different vaccine efficacies against VT and NVT, vaccination coverage, the strength of cross-protective immunity against VT and NVT are examined. The basic reproduction numbers of VT and NVT are derived from the linearlized system nearby disease-free equilibrium in the absence of the other serotype and without vaccination. Based on published estimates, *R*_0_ for EV71 are assumed to be 4.0 with the range from 1.4 to 6.5 for both VT and NVT [18], [19]. As for pneumococcus, *R*_0_ is estimated from longitudinal observation of incidence and remission [13] and are assumed to be 1.3 (range 0.9–1.4) and 1.2 (1.0–1.6) for VT and NVT, respectively. Regarding the vaccination coverage, *c*, we assume *c* = 0.5 for EV71 and *c* = 0.2 for pneumococcus, because theoretically, VT would be eliminated with higher coverage before observing the endemic steady state. The birth and death rate, *μ* is assumed to be *μ* = 1/70 per year for EV71 (crudely assuming an industrialized country) and 1/54.2 per year for pneumococcus (corresponding to the life expectancy at birth in Kenya [20]).

## Results

Figure 2 examines the sensitivity of vaccine effectiveness using the SIR model, while Figure 3 shows the effectiveness based on the SIS model. For both models and vaccine types, both VE_R_ and VE_O_ were smaller than *ε*_VT_, and VE_R_ yielded closer values to *ε*_VT_ than VE_O_. Overall, the SIR model tended to be more sensitive to vaccination coverage, the strength of naturally acquired cross-protective immunity and vaccine efficacy against NVT than SIS model does within the assumed parameter space. Regardless of the types of vaccine, SIS model appeared to yield very consistent estimates of vaccine effectiveness within the examined parameter ranges.

Differential types of vaccine generated different patterns of dependence on vaccination coverage, *c* and cross-protective immunity, *σ*. The larger *c* and *σ* are for leaky type, the greater the herd immunity and the higher both VE_R_ and VE_O_ would be. In contrast, all-or-nothing vaccine with large *c* and *σ* decreases VE_R_ and VE_O_. This is seen, because vaccinated individuals with all-or-nothing type loose the chance of infection with both VT and NVT, and as seen in equation (3), those naturally infected with NVT would consequently be reduced, leading to reduced *b*_A,AOR_ and thus, decrease in VE_R_ and VE_O_.

We have also seen remarkable dependence of vaccine effectiveness on the vaccine efficacy against NVT (Figures 2 and 3). The difference between VE_O_ and *ε*_VT_ is sensitive to vaccine efficacy against NVT (*ε*_NVT_), because VE_O_ is a function of *ε*_NVT_ as we have shown in equations (4) and (8). VE_O_ would be lowered probably by its involvement of *ε*_NVT_ in the denominator within the assumed range. Assuming that the vaccine efficacy of all-or-nothing type against VT are 70% (with the range from 60% to 80%) in the SIR model with the baseline efficacy against NVT at 30%, VE_R_ and VE_O_ are estimated at 67.8% (range: 57.4% to 78.4%) and 57.4% (43.5% to 71.3%), respectively. Employing the leaky type assumption with the SIR model, VE_R_ and VE_O_ are estimated at 53.1% (39.4% to 68.9%) and 49.0% (33.5% to 66.4%), respectively. With all-or-nothing vaccine against VT with 82% (with the range from 73% to 91%) in the SIS model along with the baseline efficacy against NVT at 10%, VE_R_ and VE_O_ are estimated at 82.0% (73.0% to 91.0%) and 78.3% (67.0% to 89.1%), respectively. Employing leaky type assumption with SIS model, VE_R_ and VE_O_ are estimated at 80.7% (72.4% to 90.3%) and 77.0% (67.1% to 88.5%), respectively.

**Figure 4:**
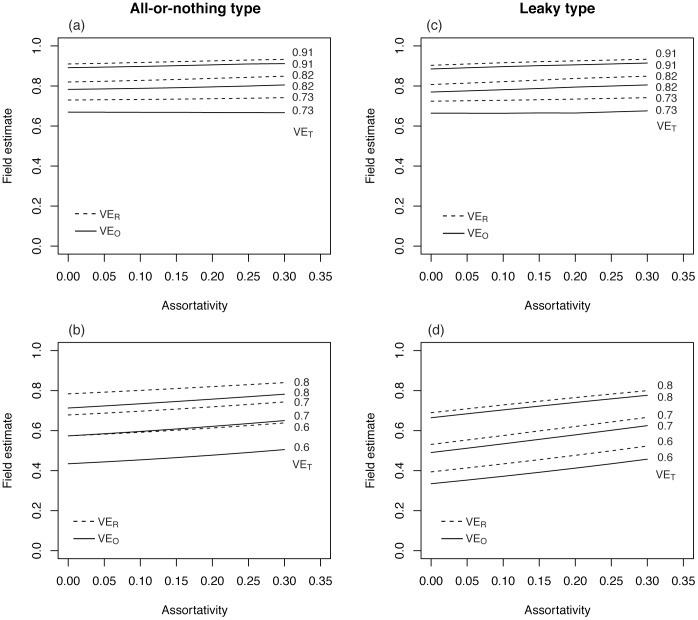
The relationship between vaccine effectiveness against VT (vaccine type) and assortativity coefficient *θ*. Solid line represents vaccine effectiveness based on odds ratio, VE_O_, while broken line represents that based on relative risk, VE_R_. Vaccine efficacy against VT is shown at the right end of lines. (a) and (c) show the result from SIR model, while (b) and (d) are from SIS model for *Streptococcus pneumoniae*. (a) and (b) show the effectiveness of all-or-nothing vaccine (i.e. perfect protection given successful immunization and no protection for unsuccessful vaccination), whereas (c) and (d) show the effectiveness of leaky vaccine (i.e. imperfect protection for all vaccinated individuals).

For both models and both types of vaccine, higher assortativity coefficients yield greater estimates of VE_R_ and VE_O_ (Figure 4). This is seen, because high assortative mixing strengthens herd immunity among vaccinated individuals. That is, high assortativity indicates that the vaccinated population is loosely connected with the unvaccinated population, and thus, the herd immunity at the subpopulation level tends to be elevated. Although equations (2) and (3) imply that the estimate of VE_R_ is always smaller than *ε*_VT_ in homogeneously mixing populations, Figure 4 indicates that the impact of heterogeneous mixing (especially that influencing the heterogeneity by vaccination status) on VE_R_ estimate is greater than the underestimation factor seen in (2) and (3).

## Discussion

The present study employed epidemiological models, investigating how vaccine effectiveness is scaled by the vaccination coverage and the two-strain transmission dynamics. Among all the results, two findings are particularly notable. First, it appeared that VE_R_ is closer to *ε*_VT_ than VE_O_, the gap of which appeared to depend on vaccine efficacy against VT (*ε*_VT_), vaccination coverage, cross-protective immunity elicited by natural infection and vaccine efficacy against NVT (*ε*_NVT_). The difference between VE_R_ and VE_O_ estimates was more apparent in the SIR model (EV71) than in the SIS model (pneumococcus). Second, among all variables of interest, model structures and assumed vaccine types, the vaccine efficacy against NVT, *ε*_NVT_, appeared to have the most profound impact on VE_O_, whereas VE_R_ was not sensitive to *ε*_NVT_. Both findings indicate that it is essential to understand the transmission dynamics of non-vaccine types so that epidemiological methods can appropriately measure the population impact of vaccination [21].

Our study was originally motivated by the need to explicitly assess the validity of indirect cohort method (Broome’s method) in which the “odds ratio of vaccination” has been used for measuring vaccine effectiveness, and thus, we expected this observation method to be very sensitive to the dynamics of NVT. In the present study, we have demonstrated that Broome’s method remains quantitatively justified under particular scenarios, e.g. especially when (i) vaccination does not protect infection with NVT and (ii) VT and NVT are not interacting from each other. Nevertheless, as can be clearly identified from the observation of serotype replacement following the introduction of PCV7 [12], it is evident that epidemiological interference exists between VT and NVT for pneumococcus [22], and the reliance of Broome’s method on NVT infections as controls has been known to be the most important pitfall in appropriately assessing the vaccine effectiveness. In the present study, we have additionally shown that VE_O_ is sensitive to vaccine efficacy against NVT. Thus, as the most important caveat, we have demonstrated that the validity of Broome’s method is highly dependent on the complex transmission dynamics including both naturally acquired immunity and vaccine-induced immunity. Fortunately, both VE_R_ and VE_O_ were far less sensitive in the SIS model to other parameters than in the SIR model, and thus, the use of Broome’s method in assessing the effectiveness of pneumococcus vaccination may in part quantitatively be justified, which echoes with a suggestion in a published study [11]. However, our exercise indicates that the validity of effectiveness estimates based on indirect cohort method should be subject to an explicit assessment by employing a prospective study design (and thus, estimating VE_R_), as long as the multi-strain dynamics and the impact of vaccination on each strain have yet to be fully quantified.

Given the potential limitation of the indirect cohort method using presently available information, what are the data gap and what one should plan to appropriately assess the vaccine effectiveness? First, the most straightforward strategy to address this issue may be to estimate VE_R_ based on a prospective study design. Of course, the cohort study requires substantial time, effort and cost, and moreover, the very low incidence of IPD is not suitable outcome for this particular design [23], and thus, one may have to consider measuring colonization rate by repeatedly isolating *S. pneumoniae* from nasal and/or throat swabs over time [24], [25]. The other aspect that requires the serious attention in future is to quantify the transmission dynamics of NVT, including interactions between VT and NVT. In particular, whereas the strength of cross-protective immunity between two different serotypes has been statistically estimated [13], one can find very few empirical estimate of the vaccine efficacy against NVT. Moreover, the dynamics involving multiple antigenic types would be more complex than we discussed here (unless symmetry in the dynamics is considered). Third, it should be noted that our discussions rest on an endemic equilibrium assumption. In reality, the epidemiological observation takes place during non-linear phase [26], [27], and theoretical and epidemiological insights into the vaccine effectiveness during such time period have yet to be closely investigated.

As a supplementary analysis, we examined how the uncertainty with respect to the model structure (i.e. SIS and SIR models) relates to the validity of measuring VE_R_ and VE_O_. As a general representation that can be interpreted as both SIS and SIR models in special cases, we constructed and analyzed the so-called SIRS (susceptible-infectious-recovered-susceptible) model with a decay rate parameter *δ*. If the rate of waning immunity *δ* is zero, the model is identical to SIR model, and as *δ*→∞, the model is interpreted as SIS model. In the online Supporting Information, we have demonstrated that the expression of VE_R_ and VE_O_ for SIRS model can be expressed as identical to what we have examined for SIS and SIR models as shown above. Numerical analysis of the SIRS model with varying *δ* has shown that the model structure has a little impact on VE_R_ and VE_O_ (Figure S1), and thus, our findings are regulated more strongly by other parameters, notably including *R*_0_. In addition, we have shown that indirect cohort method (i.e. the use of VE_O_) is sensitive to asymmetric dynamics for VT and NVT, while VE_R_ appears to be far less sensitive (Figure S2).

In summary, we have shown that the vaccine effectiveness VE_O_ calculated from odds ratio based on indirect cohort method can be vulnerable to the model type (e.g. SIR or SIS), vaccine type (e.g. all-or-nothing or leaky) and detailed mechanisms of interactions between VT and NVT. As long as the multi-strain dynamics have yet to be fully quantified, it is worthwhile to consider conducting prospective studies to estimate the VE_R_.

## Supporting Information

Figure S1 **Vaccine effectiveness in SIRS (Susceptible-Infected-Recovered-Susceptible) model.**
(TIF)

Figure S2 **Vaccine effectiveness in SIS (Susceptible-Infected- Susceptible) model.**
(TIF)

Text S1 **Supporting Information Text.**
(DOC)
