# Supplementary figures and images for: How Is Vaccine Effectiveness Scaled by the Transmission Dynamics of Interacting Pathogen Strains with Cross-Protective Immunity?
Source: PLoS One. 2012 Nov 30;7(11):e50751. doi: 10.1371/journal.pone.0050751 (PMC3511363; doi:10.1371/journal.pone.0050751)

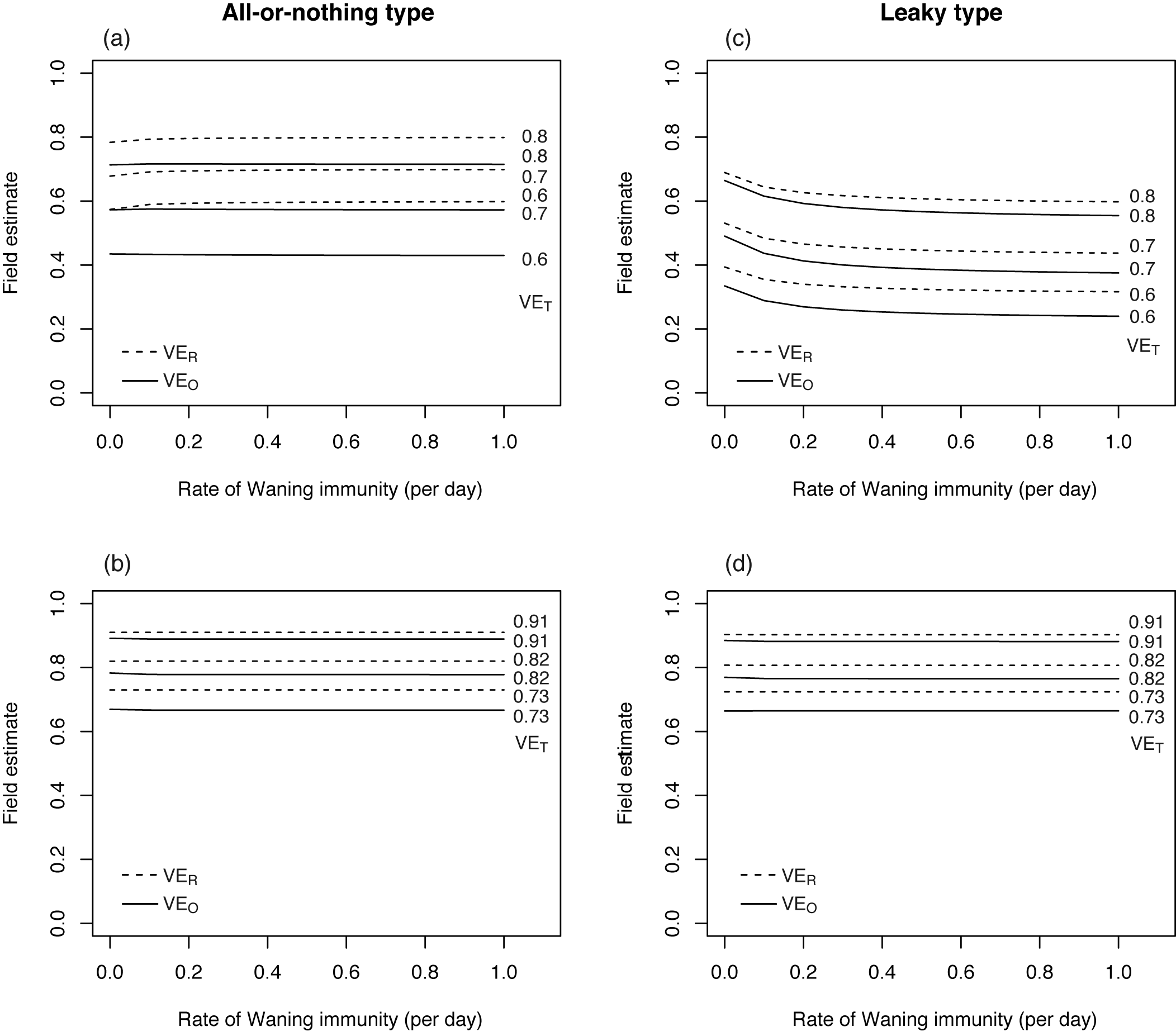

Supplement: Figure S1 — Vaccine effectiveness in SIRS (Susceptible-Infected-Recovered-Susceptible) model. (TIF) [file pone.0050751.s001.tif]

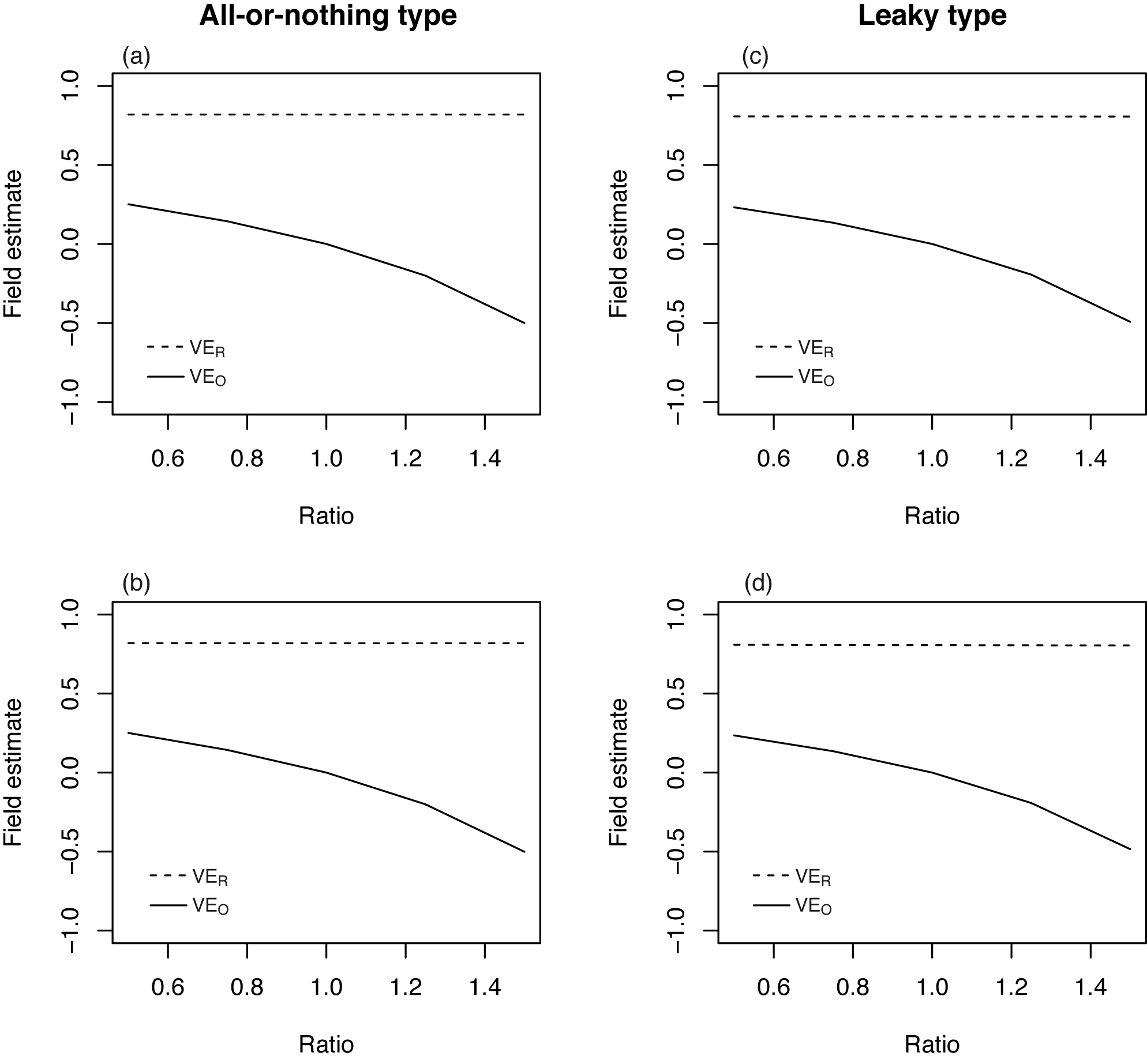

Supplement: Figure S2 — Vaccine effectiveness in SIS (Susceptible-Infected- Susceptible) model. (TIF) [file pone.0050751.s002.tif]
