# Supplementary material for: How Is Vaccine Effectiveness Scaled by the Transmission Dynamics of Interacting Pathogen Strains with Cross-Protective Immunity?
Source: PLoS One. 2012 Nov 30;7(11):e50751. doi: 10.1371/journal.pone.0050751 (PMC3511363; doi:10.1371/journal.pone.0050751)
Supplement: Text S1 — Supporting Information Text. (DOC) [file pone.0050751.s003.doc]

# **Supporting Information**

### Title: How is vaccine effectiveness scaled by the transmission dynamics of interacting pathogen strains with cross-protective immunity?

Ryosuke Omori, Benjamin J Cowling, Hiroshi Nishiura

**1. SIR model**

*All-or-nothing vaccine*

Let vaccination coverage be *c*. Let birth and death rate of host be *μ*. The time evolution of unvaccinated and vaccinated fully susceptible individuals is described by

(a1)

where *u_ss_* represents the frequency of unvaccinated (*u*) individuals susceptible to both VT (vaccine type) and NVT (non-vaccine type) (*ss*) and *v*_ss_ represents the frequency of vaccinated individuals susceptible to VT and NVT (*ss*). *λ*_A_ and *λ*_B_ are the forces (or the rates) of infection with VT and NVT, respectively, i.e.,

$\begin{matrix} \lambda_{A}=\beta_{A}(u_{is}+v_{is}+u_{ir}+v_{ir}+v_{iw}), \\ \lambda_{B}=\beta_{B}(u_{si}+v_{si}+u_{ri}+v_{ri}+v_{wi}), \end{matrix}$ (a2)

where *β*_A_ and *β*_B_ are transmission coefficients of VT and NVT, respectively. In the right-hand side of (a2), *u* and *v* represents unvaccinated and vaccinated, respectively, with subscripts, the first of which represents the state of infection with VT and the second of which with NVT (Figure 1 in the main text; *w* represents those perfectly protected by vaccination). The time evolution of those other than susceptible to both VT and NVT is described by

(a3)

where *m* denotes vaccination status (i.e. *u* if unvaccinated and *v* if vaccinated), the subscript *rc* represents the individuals recovered from VT infection and protected from NVT infection due to cross-protective immunity (and vice versa: those perfectly protected from VT infection after recovery from NVT infection by cross-protective immunity are represented by *cr*), *σ* is the relative reduction of susceptibility due to cross-protective immunity conferred by infection with the other strain (which is assumed not to be directional, i.e. infection with VT equally protects from NVT to that the infection with NVT does against VT).

The dynamics of those vaccinated and fully protected from either VT or NVT are

$\begin{matrix} v'_{sw}=\mu c(1-\varepsilon_{VT})\varepsilon_{NVT}-(\lambda_{A}+\mu)v_{sw}, \\ v'_{iw}=\lambda_{A}v_{sw}-(\gamma+\mu)v_{iw}, \\ v'_{rw}=\gamma v_{iw}-\mu v_{rw}, \\ v'_{ws}=\mu c\varepsilon_{VT}(1-\varepsilon_{NVT})-(\lambda_{B}+\mu)v_{ws}, \\ v'_{wi}=\lambda_{B}v_{ws}-(\gamma+\mu)v_{wi}, \\ v'_{wr}=\gamma v_{wi}-\mu v_{wr}. \end{matrix}$ (a4)

Considering the endemic steady state of equations (a1)-(a4), prevalence of VT at equilibrium is

$\begin{matrix} u^{*}_{is}=\frac{\lambda^{*}_{A}}{(\gamma+\mu)}u_{ss}^{*}, \\ u^{*}_{ir}=\frac{\lambda^{*}_{A}}{\gamma+\mu}(1-\sigma)u_{sr}^{*}, \\ v^{*}_{is}=\frac{c(1-\varepsilon_{VT})(1-\varepsilon_{NVT})}{(1-c)}u^{*}_{is}, \\ v^{*}_{ir}=\frac{\lambda^{*}_{A}}{\gamma+\mu}(1-\sigma)v^{*}_{sr}, \\ v^{*}_{iw}=\frac{\lambda^{*}_{A}}{(\gamma+\mu)}v^{*}_{sw}=\mu c(1-\varepsilon_{VT})\varepsilon_{NVT}\frac{\lambda^{*}_{A}}{(\lambda^{*}_{A}+\mu)}\frac{1}{(\gamma+\mu)}, \end{matrix}$ (a5)

Relative risk of infection with VT among vaccinated compared to unvaccinated, RR_VT_ is

$\mathrm{RR}_{VT}=\frac{[N(v_{is}^{*}+v_{ii}^{*}+v_{ir}^{*}+v_{iw}^{*})]/[Nc]}{[N(u_{is}^{*}+u_{ii}^{*}+u_{ir}^{*})]/[N(1-c)]}=\left(\frac{1-c}{c}\right)\left(\frac{v_{is}^{*}+v_{ii}^{*}+v_{ir}^{*}+v_{iw}^{*}}{u_{is}^{*}+u_{ii}^{*}+u_{ir}^{*}}\right),$ (a6)

where *N* describes total population size. Due to all-or-nothing vaccine, the vaccine efficacy does not alter the transmission dynamics other than reducing the size of initial susceptibles. Therefore, in equations vaccinated individuals who did not acquire immunity against both VT and NVT should be identical to unvaccinated susceptible fraction:

$\frac{v_{is}^{*}}{u_{is}^{*}}=\frac{v_{ii}^{*}}{u_{ii}^{*}}=\frac{v_{ir}^{*}}{u_{ir}^{*}}=\frac{c(1-\varepsilon_{VT})(1-\varepsilon_{NVT})}{1-c},$ (a7)

The frequency of the vaccinated individuals who did not acquire immunity against both VT and NVT and are infected with VT can be expressed with the frequency of the unvaccinated individuals who are infected with VT as follows:

$v^{*}_{is}+v^{*}_{ii}+v^{*}_{ir}=\frac{c(1-\varepsilon_{VT})(1-\varepsilon_{NVT})}{1-c}(u^{*}_{is}+u^{*}_{ii}+u^{*}_{ir}).$ (a8)

Similarly, the frequency of those at risk of infection with VT among vaccinated individuals who did not acquire immunity against both VT and NVT can be expressed with the frequency of those at risk of VT among unvaccinated individuals:

$v^{*}_{ss}+v^{*}_{si}+v^{*}_{sr}=\frac{c(1-\varepsilon_{VT})(1-\varepsilon_{NVT})}{1-c}(u^{*}_{ss}+u^{*}_{si}+u^{*}_{sr}).$ (a9)

Substituting the right-hand side of (a6) by that of equations (a8) yields

$\mathrm{RR}_{VT}=(1-\varepsilon_{VT})(1-\varepsilon_{NVT})+\left(\frac{1-c}{c}\right)\left(\frac{v_{iw}^{*}}{u_{is}^{*}+u_{ii}^{*}+u_{ir}^{*}}\right).$ (a10)

Using first, fourth and fifth sub-equations in (a5), we get

$\frac{v_{iw}^{*}}{u_{is}^{*}+u_{ii}^{*}+u_{ir}^{*}}=\frac{v_{sw}^{*}}{u_{ss}^{*}+u_{si}^{*}+u_{sr}^{*}}.$ (a11)

Using the right-hand side of equation (a6) and using the left-hand side of the first subequation of (a7), the denominator of the right-hand side of equation (a11) is rewritten as

$\frac{v^{*}_{iw}}{u^{*}_{is}+u^{*}_{ir}}=\frac{v^{*}_{sw}}{\frac{1-c}{1-c+c(1-\varepsilon_{VT})(1-\varepsilon_{NVT})}(u_{ss}^{*}+u_{si}^{*}+u_{sr}^{*}+v_{ss}^{*}+v_{si}^{*}+v_{sr}^{*})}.$ (a12)

All the derivatives of system (a3) and (a4) are zero at an endemic equilibrium. Especially, equating the derivatives of the prevalence of VT to zero, we get

$u_{ss}^{*}+u_{si}^{*}+u_{sr}^{*}+v_{ss}^{*}+v_{si}^{*}+v_{sr}^{*}+v_{sw}^{*}=\frac{\gamma+\mu}{\beta_{A}}.$ (a13)

Inserting the right-hand side of equation (a13) and $v^{*}_{sw}=\mu c(1-\varepsilon_{VT})\varepsilon_{NVT}/(\lambda^{*}_{A}+\mu)$ from equation (a4) into the denominator in the right hand-side of (a12), we obtain

$\frac{v^{*}_{iw}}{u^{*}_{is}+u^{*}_{ir}}=\frac{\{1-c+c(1-\varepsilon_{VT})(1-\varepsilon_{NVT})\}c(1-\varepsilon_{VT})\varepsilon_{NVT}}{(1-c)\left\{\frac{(\lambda^{*}_{A}+\mu)(\gamma+\mu)}{\mu\beta_{A}}-c(1-\varepsilon_{VT})\varepsilon_{NVT}\right\}}.$ (a14)

This can be used to replace the right-hand side of RR_VT_ in (a10), leading to

$\mathrm{RR}_{VT}=(1-\varepsilon_{VT})\left\{(1-\varepsilon_{NVT})+\varepsilon_{NVT}\frac{n_{ss}}{\frac{(\lambda^{*}_{A}+\mu)(\gamma+\mu)}{\mu\beta_{A}}-n_{sw}}\right\},$ (a15)

where *n*_ss_ represents the frequency of the individuals who do not possess vaccine-induced immunity (either because unvaccinated or vaccinated-and-unprotected):

$n_{ss}=1-c+c(1-\varepsilon_{VT})(1-\varepsilon_{NVT}),$ (a16)

and *n*_sw_ similarly represents the frequency of vaccinated individuals who acquired vaccine-induced immunity against NVT but did not acquire immunity against VT, which can be expressed as

$n_{sw}=c(1-\varepsilon_{VT})\varepsilon_{NVT}.$ (a17)

Equilibrium of those susceptible to VT can be considered by

$\begin{matrix} (u_{ss}+v_{ss}+u_{si}+v_{si}+u_{sr}+v_{sr}+u_{cr}+v_{cr}+v_{sw})' \\ =\mu(n_{ss}+n_{sw})-(\lambda_{A}^{*}+\mu)\{u_{ss}^{*}+v_{ss}^{*}+u_{si}^{*}+v_{si}^{*}+u_{sr}^{*}+v_{sr}^{*}+v_{sw}^{*}\}-\mu(u^{*}_{cr}+v^{*}_{cr})=0, \end{matrix}$ (a18)

from which we obtain

$\frac{\lambda^{*}_{A}+\mu}{\mu}=\frac{n_{ss}+n_{sw}-(u^{*}_{cr}+v^{*}_{cr})}{u_{ss}^{*}+v_{ss}^{*}+u^{*}_{si}+v^{*}_{si}+u_{sr}^{*}+v_{sr}^{*}+v_{sw}^{*}}.$ (a19)

Similarly, equilibrium of those infected with VT leads

$(u_{is}+v_{ir}+v_{iw})'=\lambda_{A}(u_{ss}^{*}+v_{ss}^{*}+u^{*}_{si}+v^{*}_{si}+u_{sr}^{*}+v_{sr}^{*}+v_{sw}^{*})-\frac{\lambda^{*}_{A}}{\beta_{A}}(\gamma_{A}+\mu)=0,$ (a20)

from which we obtain

$\frac{\gamma_{A}+\mu}{\beta_{A}}=u^{*}_{ss}+v^{*}_{ss}+u^{*}_{si}+v^{*}_{si}+u^{*}_{sr}+v^{*}_{sr}+v^{*}_{sw}.$ (a21)

Inserting equation (a19) and (a21) into equation (a15) yields

$\mathrm{RR}_{VT}=(1-\varepsilon_{VT})\left\{(1-\varepsilon_{NVT})+\varepsilon_{NVT}\frac{n_{ss}}{n_{ss}-(u^{*}_{cr}+v^{*}_{cr})}\right\}.$ (a22)

Accordingly, vaccine effectiveness calculated from relative risk is

$\begin{matrix} \mathrm{VE}_{R}=1-\mathrm{RR}_{VT} \\ =1-(1-\varepsilon_{VT})\left\{(1-\varepsilon_{NVT})+\varepsilon_{NVT}\frac{n_{ss}}{n_{ss}-(u^{*}_{cr}+v^{*}_{cr})}\right\}. \end{matrix}$ (a23)

Equation (a23) leads to equation (2) in the main text. Vaccine effectiveness using odds ratio of vaccination can be expressed as

$\begin{matrix} \mathrm{VE}_{O}=1-\mathrm{OR}_{VT} \\ =1-\frac{n_{v,VT}\times n_{u,NVT}}{n_{u,VT}\times n_{v,NVT}}, \end{matrix}$ (a24)

where *n*_v,VT_ represents the frequency of vaccinated individuals infected with VT, *n*_u,VT_ unvaccinated and infected with VT, *n*_v,NVT_ vaccinated and infected with NVT, *n*_u,NVT_ unvaccinated and infected with NVT, respectively. Focusing on the computational matter alone, equation (a24) suggests that VE_O_ can be calculated using the relative risks of infection with VT and NVT as

$\mathrm{VE}_{\mathrm{O}}=1-\frac{\mathrm{RR}_{VT}}{\mathrm{RR}_{NVT}}.$ (a25)

As we can similarly express RR_NVT_ as a function of *ε*_VT_, *ε*_NVT_ and other parameters, we obtain

$\mathrm{VE}_{O}=1-\left(\frac{1-\varepsilon_{VT}}{1-\varepsilon_{NVT}}\right)\frac{1+\varepsilon_{NVT}\frac{u^{*}_{cr}+v^{*}_{cr}}{n_{ss}-(u^{*}_{cr}+v^{*}_{cr})}}{1+\varepsilon_{VT}\frac{u^{*}_{rc}+v^{*}_{rc}}{n_{ss}-(u^{*}_{rc}+v^{*}_{rc})}},$ (a26)

which leads to equation (4) in the main text.

*Leaky vaccine*

In the case of leaky vaccine, instantaneous risk of infection (given an exposure) to VT (or NVT) is reduced by a factor, *ε*_VT_ (or *ε*_NVT_). The time evolution of unvaccinated and vaccinated fully susceptible individuals is described by

$\begin{matrix} u'_{ss}=\mu(1-c)-(\lambda_{A}+\lambda_{B}+\mu)u_{ss}, \\ v'_{ss}=\mu c-\left\{(1-\varepsilon_{VT})\lambda_{A}+(1-\varepsilon_{NVT})\lambda_{B}+\mu\right\}v_{ss}, \end{matrix}$ (a27)

and the time evolution of those other than susceptible to both VT and NVT is described by

$\begin{matrix} m'_{is}=a_{A,m}\lambda_{A}m_{ss}-(a_{B,m}\lambda_{B}+\gamma+\mu)m_{is}, \\ m'_{rs}=\gamma m_{is}-\{a_{B,m}(1-\sigma)\lambda_{B}+\mu\}m_{rs}, \\ m'_{si}=a_{B,m}\lambda_{B}m_{ss}-(a_{A,m}\lambda_{A}+\gamma+\mu)m_{si}, \\ m'_{ii}=a_{A,m}\lambda_{A}m_{si}+a_{B,m}\lambda_{B}m_{is}-(2\gamma+\mu)m_{ii}, \\ m'_{sr}=\gamma m_{si}-\{a_{A,m}(1-\sigma)\lambda_{A}+\mu\}m_{sr}, \\ m'_{ri}=a_{B,m}(1-\sigma)\lambda_{B}m_{rs}+\gamma m_{ii}-(\gamma+\mu)m_{ri}, \\ m'_{ir}=a_{A,m}(1-\sigma)\lambda_{A}m_{sr}+\gamma m_{ii}-(\gamma+\mu)m_{ir}, \\ m'_{rr}=\gamma(m_{ri}+m_{ir})-\mu m_{rr}, \end{matrix}$ (a28)

where *a*_A,m_ (or *a*_B,m_) is the factor of the relative reduction of susceptibility against VT (or NVT) induced by by vaccination, i.e.,

(a29)

and

(a30)

As was considered for all-or-nothing vaccine, relative risk of infection with VT leads

$\mathrm{RR}_{VT}=\frac{[N(v^{*}_{is}+v^{*}_{ii}+v^{*}_{ir})]/[Nc]}{[N(u^{*}_{is}+u^{*}_{ii}+u^{*}_{ir})]/[N(1-c)]}=\left(\frac{1-c}{c}\right)\left(\frac{v^{*}_{is}+v^{*}_{ii}+v^{*}_{ir}}{u^{*}_{is}+u^{*}_{ii}+u^{*}_{ir}}\right).$ (a31)

Considering equilibrium state of the system (a27) and (a28), relative risk is rewritten, and VE_R_ is written as

$\mathrm{VE}_{R}=1-\mathrm{RR}_{VT}=1-(1-\varepsilon_{VT})\frac{[v^{*}_{ss}+v^{*}_{si}+(1-\sigma)v^{*}_{sr}]/c}{[u^{*}_{ss}+u^{*}_{si}+(1-\sigma)u^{*}_{sr}]/(1-c)},$ (a32)

which leads to equation (6) in the main text.

**2. SIS model**

*All-or-nothing vaccine*

The time evolution of unvaccinated and vaccinated fully susceptible individuals is described by

$\begin{matrix} u'_{ss}=\mu(1-c)-(\lambda_{A}+\lambda_{B}+\mu)u_{ss}+\gamma_{A}u_{is}+\gamma_{B}u_{si}, \\ v'_{ss}=\mu c(1-\varepsilon_{VT})(1-\varepsilon_{NVT})-(\lambda_{A}+\lambda_{B}+\mu)v_{ss}+\gamma_{A}v_{is}+\gamma_{B}v_{si}, \end{matrix}$ (a33)

where *λ*_A_ and *λ*_B_ are the forces of infection of VT and NVT, i.e.,

$\begin{matrix} \lambda_{A}=\beta_{A}(u_{is}+u_{ii}+v_{is}+v_{ii}+v_{iw}), \\ \lambda_{B}=\beta_{B}(u_{si}+u_{ii}+v_{si}+v_{ii}+v_{wi}), \end{matrix}$ (a34)

The time evolution of other compartments is

(a35)

where *m* is vaccination status (unvaccinated *u* or vaccinated *v*) and *σ*_A_ (or *σ*_B_) is cross-protective immunity against VT (or NVT). The model for those who are only susceptible to VT (or NVT) and possess vaccine-induced immunity against NVT (or VT) is written as

$\begin{matrix} v'_{sw}=\mu c(1-\varepsilon_{VT})\varepsilon_{NVT}-(\lambda_{A}+\mu)v_{sw}+\gamma_{A}v_{iw}, \\ v'_{iw}=\lambda_{A}v_{sw}-(\gamma_{A}+\mu)v_{iw}, \\ v'_{ws}=\mu c\varepsilon_{VT}(1-\varepsilon_{NVT})-(\lambda_{B}+\mu)v_{ws}+\gamma_{B}v_{wi}, \\ v'_{wi}=\lambda_{B}v_{ws}-(\gamma_{B}+\mu)v_{wi}, \end{matrix}$ (a36)

Since we consider protection from IPD, i.e. severe fraction of infection, the risk measure among vaccinated is proportional to (1-*ε*_VTi_). Relative risk of IPD due to VT among vaccinated to unvaccinated is

$\mathrm{RR}_{VT}=\frac{[N(v^{*}_{is}+v^{*}_{ii}+v^{*}_{iw})(1-\varepsilon_{VTi})]/[Nc]}{[N(u^{*}_{is}+u^{*}_{ii})]/[N(1-c)]}=\frac{(1-\varepsilon_{VTi})(v^{*}_{is}+v^{*}_{ii}+v^{*}_{ic}+v^{*}_{iw})/c}{(u^{*}_{is}+u^{*}_{ii}+u^{*}_{ic})/(1-c)}.$ (a37)

As was done for SIR model, we obtain

$\begin{matrix} \mathrm{VE}_{R}=1-\mathrm{RR}_{VT} \\ =1-(1-\varepsilon_{VT})\left\{1+\varepsilon_{NVT}\frac{u^{*}_{ci}+v^{*}_{ci}}{n_{ss}-(u^{*}_{ci}+v^{*}_{ci})}\right\}. \end{matrix}$ (a38)

which leads to equation (2) in the main text.

*Leaky vaccine*

Time evolution of unvaccinated and vaccinated fully susceptible individuals is described by

$\begin{matrix} u'_{ss}=\mu(1-c)-(\lambda_{A}+\lambda_{B}+\mu)u_{ss}+\gamma_{A}u_{is}+\gamma_{B}u_{si}, \\ v'_{ss}=\mu c-\{(1-\varepsilon_{VT})\lambda_{A}+(1-\varepsilon_{NVT})\lambda_{B}+\mu\}v_{ss}+\gamma_{A}v_{is}+\gamma_{B}v_{si}, \end{matrix}$ (a39)

and time evolution of other compartment follows

(a40)

where *a*_A,m_ and *a*_B,m_ are identical to those defined in (a29) and (a30). Relative risk of IPD caused by VT among vaccinated as compared to unvaccinated leads

$\mathrm{RR}_{VT}=\frac{[N(v^{*}_{is}+v^{*}_{ir})(1-\varepsilon_{VTi})]/[Nc]}{[N(u^{*}_{is}+u^{*}_{ir})]/[N(1-c)]}=\left(\frac{1-c}{c}\right)\frac{(v^{*}_{is}+v^{*}_{ir})(1-\varepsilon_{VTi})}{u^{*}_{is}+u^{*}_{ir}}.$ (a41)

Again, as were shown above, vaccine effectiveness estimate using relative risk is

$\mathrm{VE}_{R}=1-\mathrm{RR}_{VT}=1-(1-\varepsilon_{VT})\frac{[v^{*}_{ss}+(1-\sigma_{A})v^{*}_{sr}]/c}{[u^{*}_{ss}+(1-\sigma_{A})u^{*}_{sr}]/(1-c)},$ (a42)

which leads to equation (6) in the main text. Odds ratio is considered in a similar manner to that shown for the SIR model.

**3. SIRS model**

*All-or-nothing vaccine*

The time evolution of unvaccinated and vaccinated fully susceptible individuals is described by

$\begin{matrix} u'_{ss}=\mu(1-c)-(\lambda_{A}+\lambda_{B}+\mu)u_{ss}+\delta(u_{sr}+u_{rs}), \\ v'_{ss}=\mu c-\left\{(1-\varepsilon_{VT})\lambda_{A}+(1-\varepsilon_{NVT})\lambda_{B}+\mu\right\}v_{ss}+\delta(v_{sr}+v_{rs}), \end{matrix}$ (a43)

where *λ*_A_ and *λ*_B_ are the forces (or the rates) of infection with VT and NVT, respectively,

$\begin{matrix} \lambda_{A}=\beta_{A}(u_{is}+v_{is}+u_{ii}+v_{ii}+u_{ir}+v_{ir}+v_{iw}), \\ \lambda_{B}=\beta_{B}(u_{si}+v_{si}+u_{ii}+v_{ii}+u_{ri}+v_{ri}+v_{wi}), \end{matrix}$ (a44)

The time evolution of those other than susceptible to both VT and NVT is described by

(a45)

where the subscript *rc* represents the individuals recovered from VT infection and protected from NVT infection due to cross-protective immunity (and vice versa: those perfectly protected from VT infection after recovery from NVT infection by cross-protective immunity are represented by *cr*), and *δ* denotes the rate of waning immunity. The dynamics of those vaccinated and fully protected from either VT or NVT are

$\begin{matrix} v'_{sw}=\mu c(1-\varepsilon_{VT})\varepsilon_{NVT}-(\lambda_{A}+\mu)v_{sw}+\delta v_{rw}, \\ v'_{iw}=\lambda_{A}v_{sw}-(\gamma+\mu)v_{iw}, \\ v'_{rw}=\gamma v_{iw}-(\delta+\mu)v_{rw}, \\ v'_{ws}=\mu c\varepsilon_{VT}(1-\varepsilon_{NVT})-(\lambda_{B}+\mu)v_{ws}+\delta v_{wr}, \\ v'_{wi}=\lambda_{B}v_{ws}-(\gamma+\mu)v_{wi}, \\ v'_{wr}=\gamma v_{wi}-(\delta+\mu)v_{wr}. \end{matrix}$ (a46)

Considering the endemic steady state of equations (a1)-(a4), prevalence of VT at an

As was considered for all-or-nothing vaccine, relative risk of infection with VT leads

(a47)

Considering equilibrium state of the system (a43), (a45) and (a46), relative risk is rewritten, and VE_R_ is written as

$\mathrm{VE}_{R}=1-\mathrm{RR}_{VT}=1-(1-\varepsilon_{VT})\left\{1+\varepsilon_{NVT}\frac{u^{*}_{cr}+v^{*}_{cr}}{n_{ss}-(u^{*}_{cr}+v^{*}_{cr})}\right\},$ (a48)

which leads to equation (2) in the main text. Odds ratio is calculated in the similar manner to SIS and SIR model.

*Leaky vaccine*

Time evolution of unvaccinated and vaccinated fully susceptible individuals is described by

$\begin{matrix} u'_{ss}=\mu(1-c)-(\lambda_{A}+\lambda_{B}+\mu)u_{ss}+\delta(u_{sr}+u_{rs}), \\ v'_{ss}=\mu c-\left\{(1-\varepsilon_{VT})\lambda_{A}+(1-\varepsilon_{NVT})\lambda_{B}+\mu\right\}v_{ss}+\delta(v_{sr}+v_{rs}), \end{matrix}$ (a49)

and time evolution of other compartment follows

$\begin{matrix} m'_{is}=a_{A,m}\lambda_{A}m_{ss}-(a_{B,m}\lambda_{B}+\gamma+\mu)m_{is}+\delta m_{ir}, \\ m'_{rs}=\gamma m_{is}-\{\delta+a_{B,m}(1-\sigma)\lambda_{B}+\mu\}m_{rs}+\delta m_{rr}, \\ m'_{si}=a_{B,m}\lambda_{B}m_{ss}-(a_{A,m}\lambda_{A}+\gamma+\mu)m_{si}+\delta m_{ri}, \\ m'_{sr}=\gamma m_{si}-\{\delta+a_{A,m}(1-\sigma)\lambda_{A}+\mu\}m_{sr}+\delta m_{rr}, \\ m'_{ri}=a_{B,m}(1-\sigma)\lambda_{B}m_{rs}+\gamma m_{ii}-(\delta+\gamma+\mu)m_{ri}, \\ m'_{ir}=a_{A,m}(1-\sigma)\lambda_{A}m_{sr}+\gamma m_{ii}-(\delta+\gamma+\mu)m_{ir}, \\ m'_{ii}=a_{A,m}\lambda_{A}m_{si}+a_{B,m}\lambda_{B}m_{is}-(2\gamma+\mu)m_{ii}, \\ m'_{rr}=\gamma(m_{ri}+m_{ir})-(2\delta+\mu)m_{rr}. \end{matrix}$ (a50)

Relative risk of IPD caused by VT among vaccinated as compared to unvaccinated leads

(a51)

Similar as SIS and SIR model, vaccine effectiveness estimate using relative risk is

$\mathrm{VE}_{R}=1-\mathrm{RR}_{VT}=1-(1-\varepsilon_{VT})\frac{[v^{*}_{ss}+v^{*}_{si}+(1-\sigma_{A})v^{*}_{sr}]/c}{[u^{*}_{ss}+u^{*}_{si}+(1-\sigma_{A})u^{*}_{sr}]/(1-c)},$ (a52)

which leads to equation (6) in the main text. Odds ratio is calculated by same manner in SIS and SIR model.

**4. The impact of asymmetric vaccine efficacies on the vaccine effectiveness**

In the main text, we discussed the dynamics of *Streptococcus pneumoniae* using asymmetric (i.e. unequal) parameter values between VT and NVT, because of empirical indications of the asymmetric epidemiological dynamics by antigenic types. To understand how the asymmetry of parameters influences the vaccine effectiveness, we focused on asymmetry in vaccine efficacy and estimated the vaccine effectiveness by different levels of asymmetry in vaccine efficacies between VT and NVT using symmetric assumption for all other parameters.

Supplementary Figure 2 examines the sensitivity of VE to different ratios of vaccine efficacy against NVT to efficacy against VT, *ε*_NVT_/*ε*_VT_. To ensure comparativeness and to gain theoretical insights into the impact of asymmetry on vaccine effectiveness, the prevalence of the total population was kept constant. VE_R_ did not appear particularly sensitive to asymmetry of vaccine efficacy between VT and NVT. However, VE_O_ greatly varied with asymmetry of vaccine efficacy. The finding here indicates a need to explore the impact of asymmetric parameters on the multistrain transmission dynamics including the effectiveness of strain specific interventions. Such a question also extends to the difficulty in comparatively assessing the role of asymmetry in the transmission dynamics during computations.


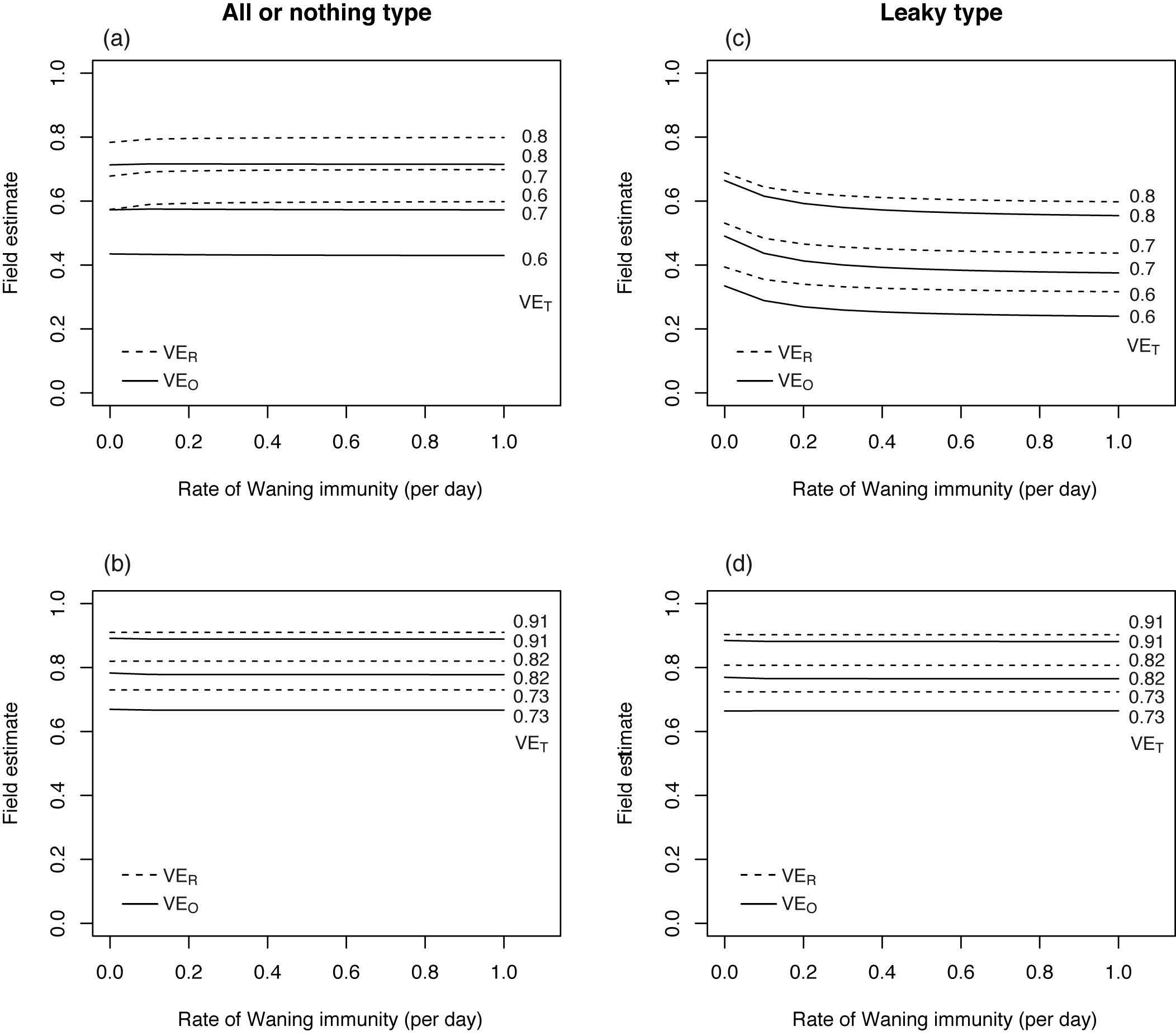


Supplementary Figure 1. Vaccine effectiveness in SIRS (Susceptible-Infected-Recovered-Susceptible) model. Field estimate (vertical axis) represents the vaccine effectiveness estimate derived from empirical observation in the field. Solid line represents vaccine effectiveness based on odds ratio, VE_O_, while broken line represents that based on relative risk, VE_R_. Assumed vaccine efficacy against VT (vaccine type) is shown at the right end of each line. (a) and (b) show the effectiveness of all-or-nothing vaccine and cross-protective immunity (i.e. perfect protection given successful immunization and no protection for unsuccessful vaccination), whereas (c) and (d) show the effectiveness of leaky vaccine (i.e. imperfect protection for all vaccinated individuals). (a) and (c) shows the vaccine effectiveness with the same parameter set as the baseline of EV71. (b) and (d) shows the vaccine effectiveness with the same parameter set as the baseline of *Streptococcus pneumoniae*.


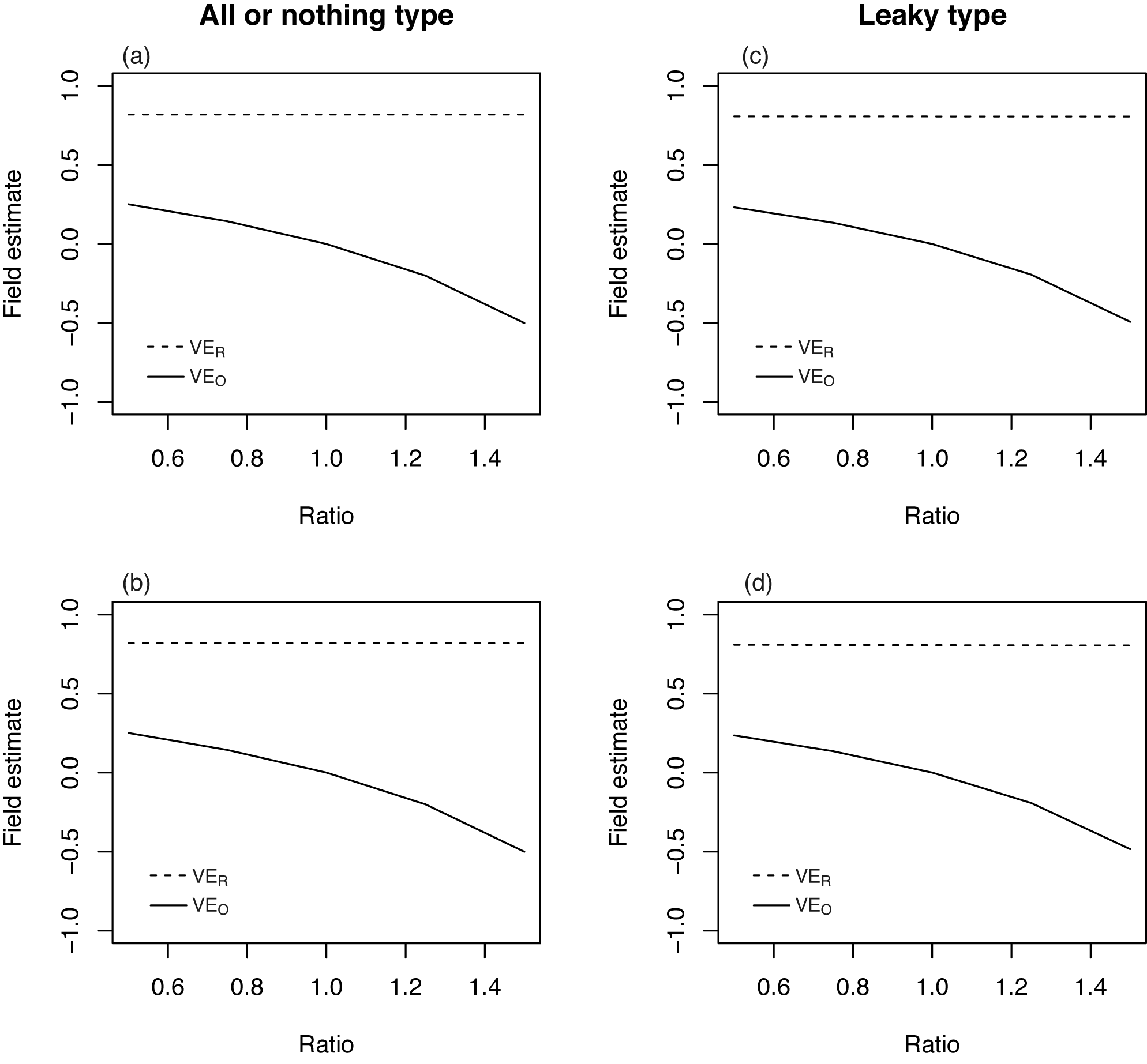


Supplementary Figure 2. Vaccine effectiveness in SIS (Susceptible-Infected- Susceptible) model.

Field estimate (vertical axis) represents the vaccine effectiveness derived from empirical observation in the field. Solid line represents vaccine effectiveness based on odds ratio, VE_O_, while broken line represents that based on relative risk, VE_R_. The horizontal axis “ratio” represents the ratio of vaccine efficacy against NVT to the efficacy against VT, *ε*_NVT_/*ε*_VT_. Namely, the ratio being 1 represents symmetric efficacy, while others are asymmetric. Assumed vaccine efficacy against VT (1-(1-*ε*_VT_) (1-*ε*_VTi_)) is fixed at 0.82, and *ε*_NVT_ is varied. All other parameters are kept symmetric between VT and NVT (i.e. *R*_0,VT_=*R*_0,NVT_=1.3, 1/*γ*_A_=1/*γ*_B_=50 days, *σ*_A_=*σ*_B_=0.1 and *ε*_VTi_=*ε*_NVTi_=0.7). (a) and (b) show the effectiveness with all-or-nothing vaccine, whereas (c) and (d) show the effectiveness with leaky vaccine. (a) and (c) show the vaccine effectiveness with different *R*_0_ while keeping the prevalence of VT a constant. (b) and (d) show the vaccine effectiveness while maintaining the prevalence of the sum of VT and NVT a constant.
